# Retraction: TiO_2_ Nanoparticles Induced Hippocampal Neuroinflammation in Mice

**DOI:** 10.1371/journal.pone.0297256

**Published:** 2024-01-11

**Authors:** The PLOS ONE Editors

Following the publication of this article [1], multiple concerns were raised regarding the results and text. Specifically,

- Values in Tables 2 and 3 described as standard deviation (SD) consistently represent 5% of the mean.
- There is text overlap between the Discussion section of [1] and previously published articles with no shared authors [2, 3], without attribution.
- There is text overlap between the Methods and Results sections of [1] and other works by the same author group that were published first [4, retracted in 5, 6, 7]. Text reuse was not cited or acknowledged in [1] and similar submitted manuscripts were not declared during the peer review process as is required by PLOS policy.
- Error bars in Figures 4, 5, 7 and 8 do not appear uniform, and it is not clear if these error bars represent SD as described in the legends or if they were calculated as 5% of the mean as in the tables.
- The numbers of animals reported in the Methods section and Results do not align.

The corresponding author stated that an error occurred in the calculation of SD values. They provided updated tables presenting mean ± standard deviation, as well as raw numerical data underlying the results presented in these tables.

The author also stated that one-way ANOVA was used to compare groups in the tables, however they did not clarify if a post-hoc test was used to compare individual groups, and overall the statistical analysis methods are not adequately reported.

In light of the above issues, the article does not meet the journal’s reporting and policy requirements and PLOS remains concerned about the validity and reliability of the reported quantitative results. Therefore, the *PLOS ONE* Editors retract this article. We regret that the issues were not identified before the article was published.

All authors either did not respond directly to the editorial decision or could not be reached.
